# Deep learning for accurate classification of conifer pollen grains: enhancing species identification in palynology

**DOI:** 10.3389/fdata.2025.1507036

**Published:** 2025-02-14

**Authors:** Masoud A. Rostami, LeMaur Kydd, Behnaz Balmaki, Lee A. Dyer, Julie M. Allen

**Affiliations:** ^1^Data Science Division, University of Texas at Arlington, Arlington, TX, United States; ^2^Department of Biology, University of Texas at Arlington, Arlington, TX, United States; ^3^Department of Biological Sciences, Virginia Tech, Blacksburg, VA, United States; ^4^Department of Biology, University of Nevada, Reno, NV, United States

**Keywords:** deep learning, ecological research, environmental changes, palynology, transfer learning

## Abstract

Accurate identification of pollen grains from *Abies* (fir), *Picea* (spruce), and *Pinus* (pine) is an important method for reconstructing historical environments, past landscapes and understanding human-environment interactions. However, distinguishing between pollen grains of conifer genera poses challenges in palynology due to their morphological similarities. To address this identification challenge, this study leverages advanced deep learning techniques, specifically transfer learning models, which are effective in identifying similarities among detailed features. We evaluated nine different transfer learning architectures: DenseNet201, EfficientNetV2S, InceptionV3, MobileNetV2, ResNet101, ResNet50, VGG16, VGG19, and Xception. Each model was trained and validated on a dataset of images of pollen grains collected from museum specimens, mounted and imaged for training purposes. The models were assessed on various performance metrics, including accuracy, precision, recall, and F1-score across training, validation, and testing phases. Our results indicate that ResNet101 relatively outperformed other models, achieving a test accuracy of 99%, with equally high precision, recall, and F1-score. This study underscores the efficacy of transfer learning to produce models that can aid in identifications of difficult species. These models may aid conifer species classification and enhance pollen grain analysis, critical for ecological research and monitoring environmental changes.

## 1 Introduction

The scientific study of pollen is a key step in studies of historical and contemporary environmental analysis. Researchers use pollen data to reconstruct past vegetation patterns and understand changes in landscapes in paleoecology and climate change studies (Willard and Bernhardt, 2011; Shennan, 2015; Balmaki et al., 2019, 2024). By examining pollen grains preserved in sediments or peat bogs, paleoecologists can identify the types of vegetation that existed in a particular area at different times in the past. This information is used to help reconstruct historical climate conditions, as the distribution of plants is closely linked to specific climate parameters such as temperature and rainfall. Through this method, scientists can trace the ecological impacts of climate fluctuations over centuries, providing insights into how ecosystems responded to changes in the environment and aiding predictions of future ecological responses to climate change (Balmaki et al., 2019). Moreover, pollen grains can help to identify the interactions between human activities and environmental factors that significantly shape these landscape patterns (Sadori et al., 2010; Kobe et al., 2020).

Coniferous genera, in particular, are representative of specific ecological and climatic adaptations making them important to help map past landscapes and climate conditions. For example, fir trees (*Abies*) are sensitive to moisture changes, spruce trees (*Picea*) are adapted to cold environments, and pine trees (*Pinus*) are known to be resilient to various environmental stresses including fire. Collectively, data on these taxa provide a comprehensive understanding of historical humidity, precipitation patterns, climatic fluctuations, and fire regimes (Latałowa and van der Knaap, 2006; Balmaki and Wigand, 2019; Larson et al., 2020). Beyond its significance in reconstructive studies, pollen analysis, drawing from these conifers, is also critical to health and allergy research, helping to identify allergenic species and predict pollen-related health issues, thus playing a key role in public health management and allergen treatment (Gastaminza et al., 2009; Frisk et al., 2024). Traditional palynology, the study of pollen grains and spores, depends on morphological characters of pollen grains to identify taxa. Typical traits include shape, polarity, symmetry, apertures, size, and ornamentation. However, the subtle morphological differences between closely related pollen grains make it challenging to distinguish species accurately and quickly. Identifying pollen grains under the microscope is time-consuming, expensive, and dependent on subjective criteria, resulting in error rates as high as 33% (Langford et al., 1990; Gonçalves et al., 2016; Sevillano et al., 2020). Although digital imaging techniques and graphical software have been used to enhance analysis, these tasks largely rely on human visual inspection, which is prone to classification errors, particularly for novice palynologists. Such limitations highlight the need for more efficient, objective, and accurate methods of pollen identification.

**Figure 1:**
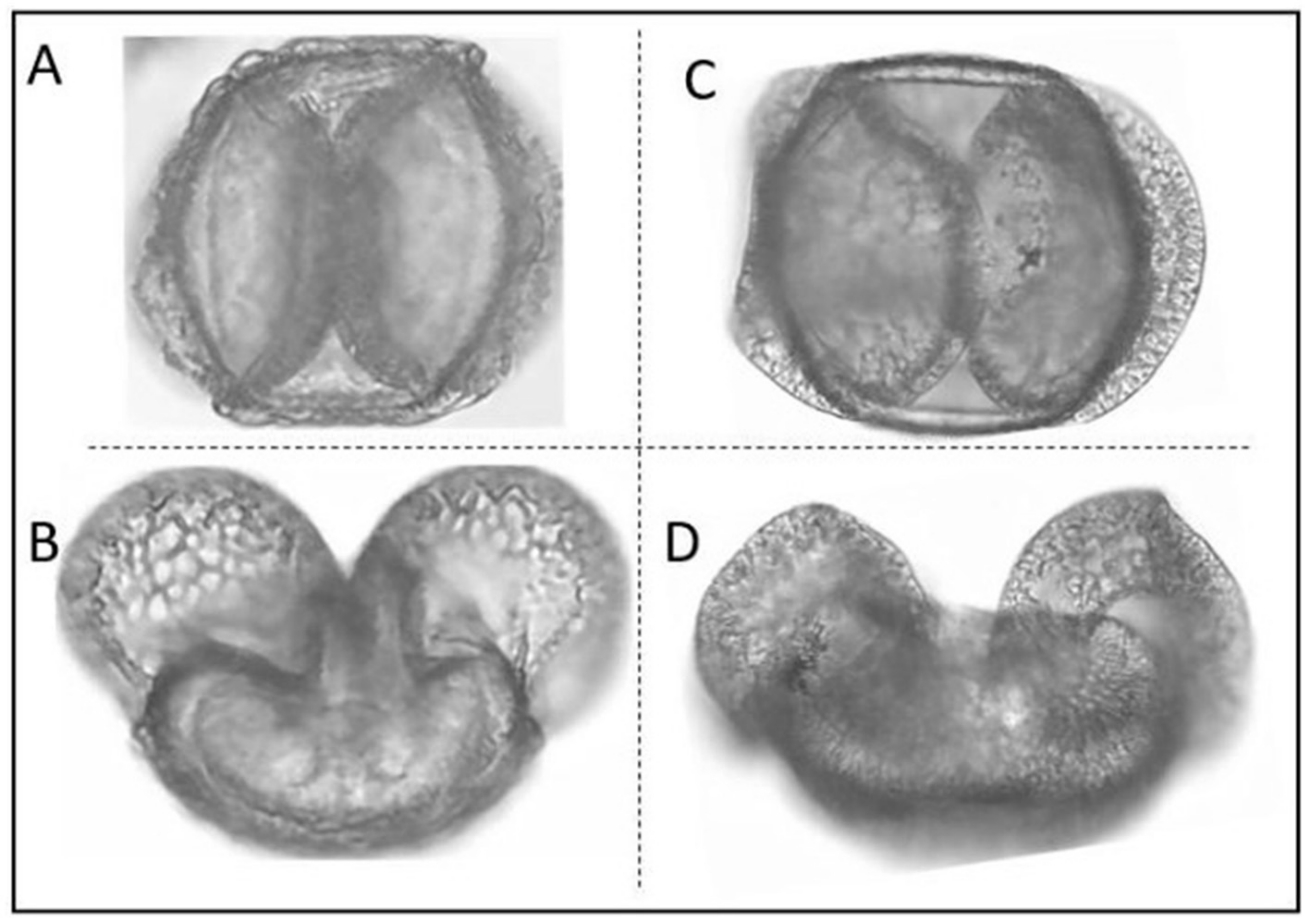
Morphological Comparison of *Pinus monophylla* **(A, B)** and *Abies concolor* **(C, D)**. In this image each grain is visualized from two different angles. Conifer species tend to have morphologically similar pollen grains making their identification difficult.

Identification of pollen grains is a difficult task, requiring both expert knowledge and high-resolution micrographs as well as a large number of reference slides for accurate comparison and identification. In particular, pollen grains of conifer species such as fir, spruce and pine, are difficult to identify because they all have two air sacs with a central body, causing the grains of these groups to look very similar with little morphological distinctness (Figure 1). This issue has been extensively documented, notably in a detailed study by Bagnell (1975), where distinctions among several species of Abies, Picea, and Pinus were meticulously examined using scanning electron microscopy. This study highlighted the subtle morphological differences critical for species identification, reinforcing observations from other research that document the morphological overlap among these species and the consequent challenges in their microscopic identification. Due to these morphological similarities, accurately identifying these species under a microscope is challenging. Deep learning is a great technique for enhancing pollen analysis by identifying species from a model trained on thousands of images (e.g., Daood et al., 2016). Having a trained model may help to improve our identification of these species and reduce the need for extensive morphological training in palynology. Deep learning approaches not only improve the accuracy of pollen classification but also dramatically reduce the time required for identification compared to traditional methods. While manual identification of a single pollen sample may take several minutes to hours depending on expertise and sample complexity, ML/DL models can process thousands of images in seconds once trained, providing an exponential improvement in speed (Balmaki et al., 2022b; Rostami et al., 2023). This makes deep learning particularly valuable for large-scale ecological and environmental studies.

In this study, we examine the ability of deep learning to identify pollen grains from conifer species. Deep learning techniques enhance accuracy, efficiency, and reduce manual effort and errors across image classification, object detection, and task recognition, as evidenced by multiple studies (Wäldchen and Mäder, 2018; Buddha et al., 2019; Afonso et al., 2020; Norouzzadeh et al., 2021; Jabbar et al., 2024). Specifically, deep learning has been highly effective in pollen taxonomic classification, utilizing transfer learning to achieve notable advancements (Daood et al., 2016; Khanzhina et al., 2018; Sevillano and Aznarte, 2018; Sevillano et al., 2020; Jaccard et al., 2020; Polling et al., 2021; Olsson et al., 2021; Zeng et al., 2021; Balmaki et al., 2022b; Rostami et al., 2023). Transfer learning, a technique where a model developed for one task is adapted for another, is particularly valuable as it leverages pre-trained models on large, diverse datasets to enhance learning efficiency and prediction accuracy, even with limited data specific to conifer species. This approach is cost-effective and less time-consuming, addressing the challenges of data scarcity in this domain.

## 2 Materials and methods

### 2.1 Data collection and pollen analysis

For this research, we selected six common pollen species from the Pinaceae family: *Abies concolor, Picea pungens, Picea wilsonii, Pinus flexilis, Pinus monophylla*, and *Pinus sabiniana*, all sourced from the University of Nevada, Reno Museum of Natural History (UNRMNH). We manually collected pollen grains from the herbarium collections of a historical museum using entomological pins under a binocular microscope. We checked the pollen grains to ensure that no contaminant grains were present (e.g., grains from other plant species, potentially brought in by wind or insects). Pollen grains were prepared on glass slides by applying two drops of 2,000 cs silicone oil. This suspension allowed for the pollen grains to be rotated under the microscope, making it easier to examine their dimensions and shapes from various angles. All pollen grains are arranged on the slide to prevent them from sticking together, making it easier to take pictures for creating models. Each slide contained at least 400 individual pollen grains. Slides were secured with cover slips and sealed with nail polish as in Balmaki et al. (2019, 2022a, 2022b). A ZEISS Axiolab 5 light microscope paired with an Axiocam 208 color microscope camera was used to photograph the pollen grains. Images were taken using 20 × objective lenses and 10 × ocular lenses. We collected a dataset of nearly 1,400 images of pollen grains. The dataset includes images from the six pollen species, with each class containing between 96 and 487 images. All images were standardized to a resolution of 224 × 224 pixels and saved in JPEG format. For further details, see Table 1.

**Table 1:** Distribution of pollen grain images across different species and percentage of images each species had in the model.

**Image classes**	**No. of Images**	**Percentage (%)**
*Abies concolor*	115	8.5
*Picea pungens*	292	21.6
*Picea wilsoni*	264	19.5
*Pinus flexilis*	99	7.3
*Pinus monophylla*	96	7.1
*Pinus sabiniana*	487	36
Total	1,353	100

### 2.2 Data preprocessing

**Figure 2:**
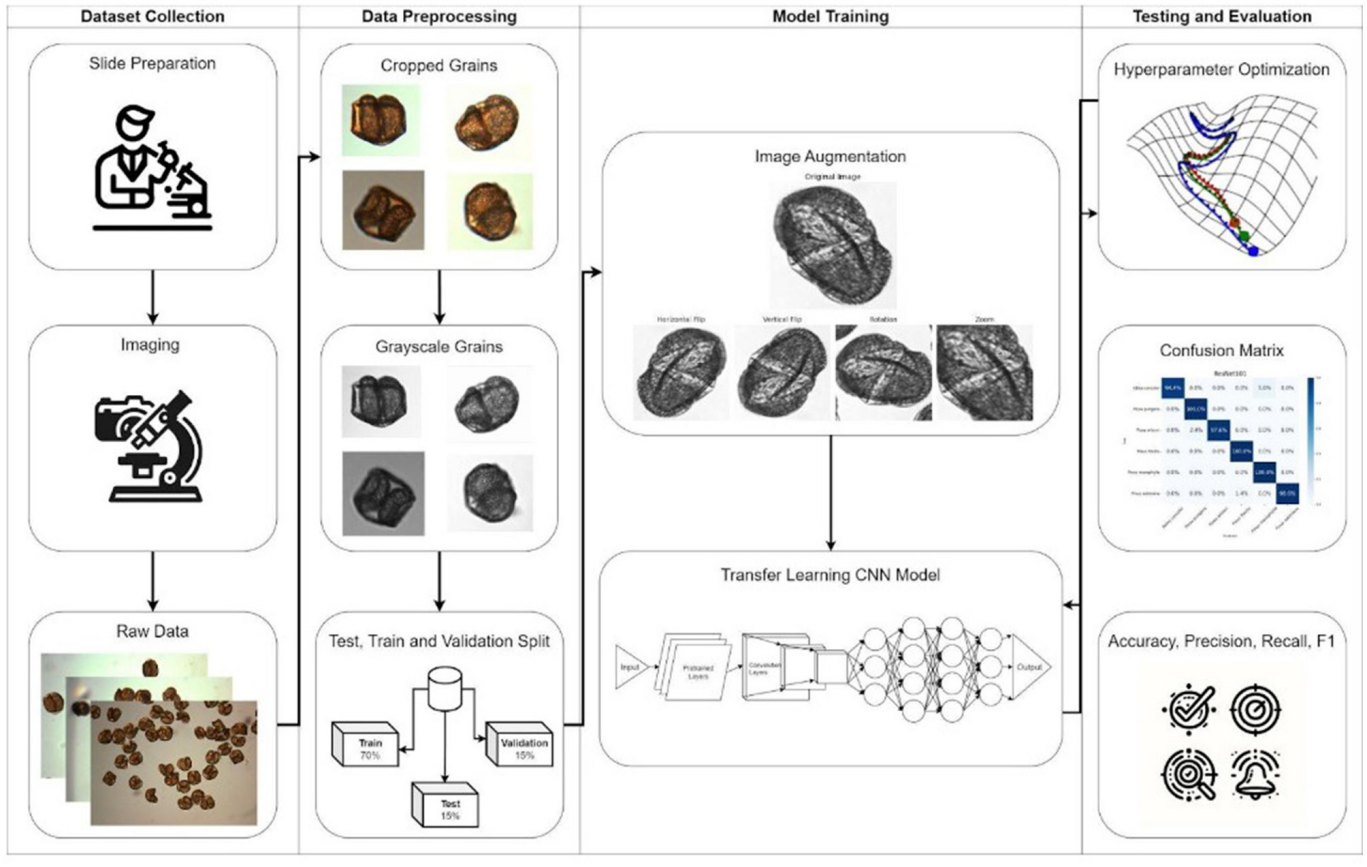
Methodological Pipeline for Pollen Analysis: This figure illustrates the comprehensive process used for pollen data analysis, starting from dataset collection through slide preparation and imaging. It details data preprocessing steps including cropping and converting pollen grains to grayscale, followed by a split into training, validation, and test sets. The model training phase incorporates image augmentation techniques to enhance the robustness of the transfer learning CNN model. The final evaluation phase is depicted through different metrics, including a confusion matrix to illustrate the model's performance.

**Figure 3:**
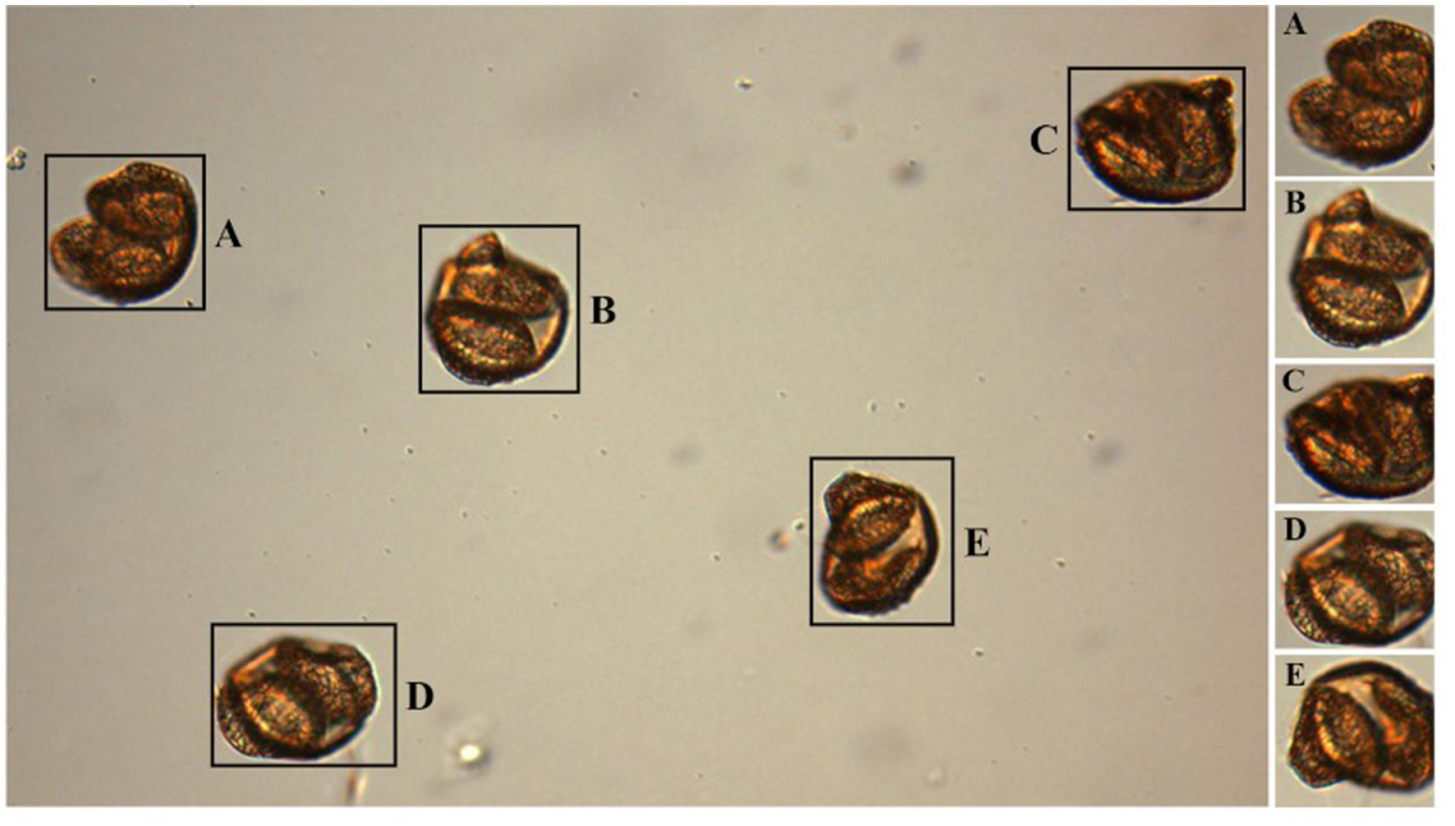
Cropped Pollen Grains from Microscopic Imaging: This figure displays individual pollen grains **(A–E)** extracted from a single slide. The cropping process isolates each grain for detailed analysis and further study in pollen classification.

The methodology pipeline is illustrated in Figure 2, encompassing the stages of dataset collection, data preprocessing, model training with image augmentation, and evaluation through hyperparameter optimization and performance metrics. For model training, each image was cropped to ensure consistency and to focus on relevant details. We used the Python-based OpenCV (Version 4.9) package for segmenting images containing multiple pollen grains into individual images and then converting to grayscale. Thresholding and morphological operations were then applied to highlight and clean up the particles within the images. Contours of these particles were identified and filtered based on a specified diameter range to avoid segmenting particles that were either too small, which could be dust or bubbles, or too large, which might result from overlapping grains creating an abnormally large particle. For each particle that met the criteria, the region of interest was cropped with an added margin, and the resulting cropped images were saved (Figure 3). Challenges such as overlapping grains and misidentified dust particles required manual exclusion from the final dataset.

Before training the models, the dataset underwent comprehensive preprocessing. This included normalizing pixel values within a specified range and resizing all images to align with model input requirements. To increase model robustness and reduce the likelihood of overfitting, data augmentation techniques such as random rotations, flipping, and zooming were utilized. The complete dataset consisted of 1,344 images and was systematically divided into three subsets: training, testing, and validation. The training subset contained 70% of the total images (944 images), while the testing and validation subsets each comprised 15%, with 200 images per subset. This structure was chosen to optimize the effectiveness of model training.

### 2.3 Transfer learning architectures

We selected these models based on their varied architectures and proven effectiveness in image recognition tasks, adapting each to our dataset's unique requirements through a systematic process of feature adjustment and fine-tuning. In this study, we tested nine different transfer learning models to tackle the challenge of distinguishing similar pollen grains from *Abies, Picea*, and *Pinus* species. Each of these models—DenseNet201, EfficientNetV2S, InceptionV3, MobileNetV2, ResNet101, ResNet50, VGG16, VGG19, and Xception has been pre-trained on large-scale image datasets, making them well-suited for feature extraction in complex image recognition tasks. The use of multiple models allows us to compare their effectiveness and robustness across similar images, ensuring that we can identify the most effective architecture for our specific application.

Each model employed a combination of feature transfer, parameter transfer, and layer fine-tuning. The convolutional bases of each model were utilized as fixed feature extractors where only the top layers were retrained to adapt to the nuances of our pollen dataset. Parameters from select layers were finely adjusted to better suit the detailed features of pollen grains. This adaptation was crucial for enhancing the model's sensitivity to subtle inter-species variations. After the initial adaptation phase, several layers were progressively unfrozen and fine-tuned with a reduced learning rate to allow precise adjustments, optimizing the models for high accuracy in pollen classification.

#### 2.3.1 DenseNet201

This architecture utilizes dense connections, where each layer receives inputs from all preceding layers and passes on its feature-maps to subsequent layers. This effectively reduces the vanishing gradient problem, enhances feature propagation, and facilitates feature reuse. This design simplifies training and increases parameter efficiency by ensuring that each layer can access feature maps from every other layer, enhancing the learning and feature-utilization efficiency (Huang et al., 2017).

#### 2.3.2 EfficientNetV2S

This architecture employs a compound scaling method that uniformly scales the depth, width, and resolution of the network. This approach optimizes both accuracy and efficiency, reducing training time and improving scalability across various devices. Fine-tuning was particularly focused on the scaling parameters to match the complexity of pollen images (Tan and Le, 2021).

#### 2.3.3 InceptionV3

This architecture builds on its predecessor, InceptionV2, by incorporating factorization into its modules to reduce computational load. It uses asymmetric convolutions that split larger convolutions into smaller ones, effectively reducing operations and complexity. This optimization enhances performance in large-scale image recognition tasks and was fine-tuned to improve the model's capacity to process the unique textural features of pollen (Szegedy et al., 2016).

#### 2.3.4 MobileNetV2

This architecture introduces inverted residuals and linear bottlenecks with shortcut connections that maintain a compact and efficient model structure, ideal for mobile environments with limited computational resources. This model was adapted for rapid processing, enabling efficient deployment in field studies (Sandler et al., 2018).

#### 2.3.5 ResNet series (ResNet50 and ResNet101)

The ResNet series enhances deep learning architectures using deep residual frameworks. These models use skip connections to facilitate training deeper networks by preventing vanishing gradients and degradation. ResNet50 offers a balance between performance and computational cost, whereas ResNet101 provides greater depth for enhanced feature learning, crucial for distinguishing closely similar pollen types (He et al., 2016).

#### 2.3.6 VGG series (VGG16 and VGG19)

The VGG series standardizes deep learning architectures using repetitive 3 × 3 convolutions and pooling layers. VGG16 focuses on building rich feature hierarchies' layer by layer, while VGG19, an extension with more convolutional layers, captures more complex features, benefiting tasks requiring detailed feature differentiation and was particularly useful in identifying subtle morphological differences between pollen species (Simonyan and Zisserman, 2014).

#### 2.3.7 Xception

This architecture refines Inception by introducing depthwise separable convolutions, decoupling the learning of spatial hierarchies from channel-wise correlations. This improvement boosts both performance and efficiency, making it ideal for handling large-scale, high-dimensional data and was adapted to enhance feature extraction capabilities specific to the textural and shape-related characteristics of different pollen grains (Chollet, 2017).

### 2.4 Experimental design and optimization techniques

All models were trained and tested on an NVIDIA GeForce RTX 3060 with 12GB of memory using Python 3.10.6 and TensorFlow. Each model was configured with a learning rate of 0.001, a batch size of 128, and the number of epochs adjusted between 30 and 100 to match the complexity and convergence behavior of each model. The total parameters and disk storage varied significantly across models, for instance, DenseNet201 had ~96 million parameters with a disk size of 369.53 MB, while EfficientNetV2S utilized about 131 million parameters with a disk size of 500.05 MB. This design and approach were critical for reliable and efficient training, essential for precise classification of closely similar pollen species. Table 2 provides an overview of the hyperparameter configurations, detailing the specific settings for each model. This includes the use of pre-trained ImageNet weights, the ADAM optimizer, and early stopping mechanisms (implemented if no improvement is observed), with the loss function set to sparse categorical cross entropy. The variation in the number of epochs and the strategic implementation of early stopping are tailored to optimize each model's learning process effectively.

**Table 2:** Overview of hyperparameter configurations for various transfer learning models.

**Model name**	**Learning rate**	**Optimizer**	**Early stopping**	**Epochs**	**Batch size**
DenseNet201	0.001	ADAM	10	48	128
EfficientNetV2S	0.001	ADAM	10	100	128
InceptionV3	0.001	ADAM	10	54	128
MobileNetV2	0.001	ADAM	10	65	128
ResNet101	0.001	ADAM	10	54	128
ResNet50	0.001	ADAM	10	49	128
VGG16	0.001	ADAM	20	30	128
VGG19	0.001	ADAM	20	57	128

## 3 Results and discussions

**Figure 4:**
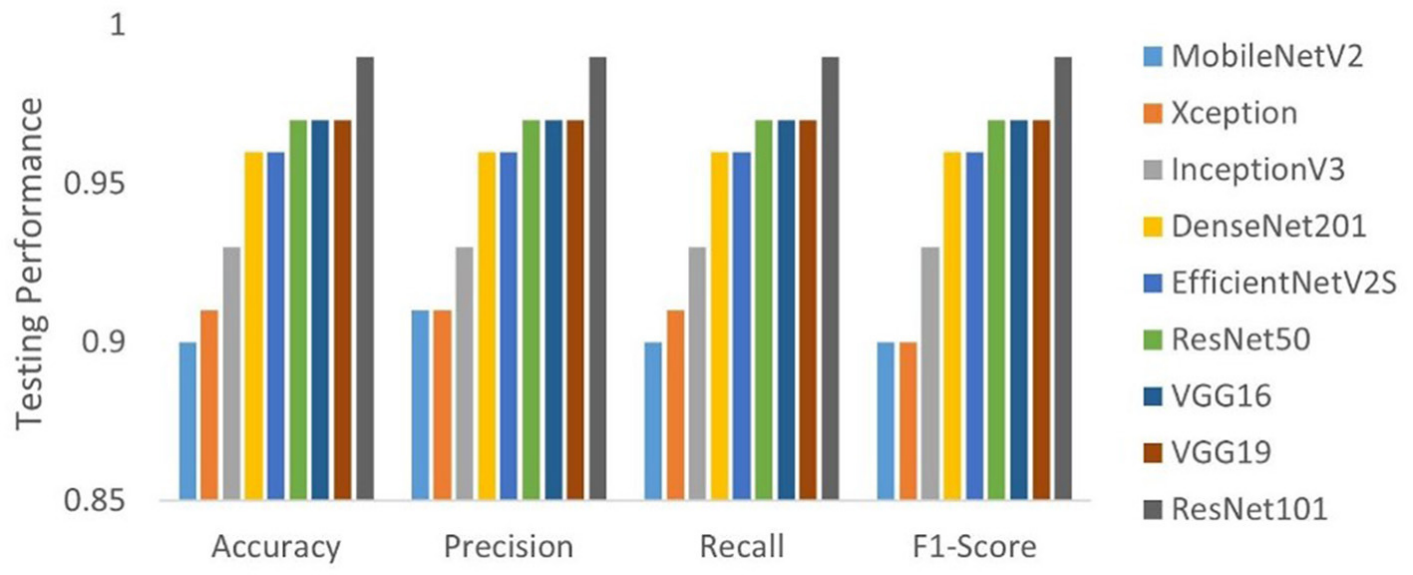
Comparative testing performance of the deep learning models tested.

In our evaluation of various transfer learning models for the classification of similar pollen grains from *Abies, Picea*, and *Pinus* species, the ResNet models, particularly ResNet50 and ResNet101, outperformed the other models (Table 3). The confusion matrix for ResNet101 had the highest accuracy in pollen classification, with minimal misclassifications (Figure 4). This performance can be attributed primarily to the architectural advantages of the ResNet models. ResNet architectures, such as ResNet50 and ResNet101, are particularly effective for tasks like conifer species classification due to their deep residual learning framework (He et al., 2016). This framework mitigates the vanishing gradient problem through the use of skip connections that facilitate direct gradient flow across multiple layers. This enhancement not only accelerates training but also improves learning capabilities as network depth increases, which is essential for distinguishing subtle features in highly similar classes. The effectiveness of these models is demonstrated by the high-performance metrics, as detailed in Table 1, with ResNet101 achieving nearly perfect scores in accuracy, precision, recall, and F1-score. A key strength of ResNet models is their scalability, which allows them to effectively manage hundreds of layers without performance degradation. This attribute is key for addressing the challenges posed by high intra-class variation and subtle inter-class differences observed in pollen grain images (He et al., 2016).

**Table 3:** Comparison of model performance metrics across various transfer learning models used in this study.

**Model name**	**Training**		**Validation**		**Testing**	**Other metrics**		
	**Accuracy**	**Loss**	**Accuracy**	**Loss**	**Accuracy**	**Precision**	**Recall**	**F1-Score**
DenseNet201	0.9576	0.1176	0.945	0.2066	0.96	0.96	0.96	0.96
EfficientNetV2S	0.9544	2.206	0.965	2.1986	0.96	0.96	0.96	0.96
InceptionV3	0.9057	0.2278	0.880	0.3492	0.93	0.93	0.93	0.93
MobileNetV2	0.8972	1.7904	0.850	1.9488	0.90	0.91	0.90	0.90
ResNet101	0.9725	0.1051	0.980	0.0989	0.99	0.99	0.99	0.99
ResNet50	0.9915	0.0273	0.990	0.045	0.97	0.97	0.97	0.97
VGG16	0.9756	0.0717	0.965	0.1089	0.97	0.97	0.97	0.97
VGG19	0.9873	0.0446	0.995	0.0645	0.97	0.97	0.97	0.97

The rows highlighted in green represent the top two models with the highest performance metrics.

**Figure 5:**
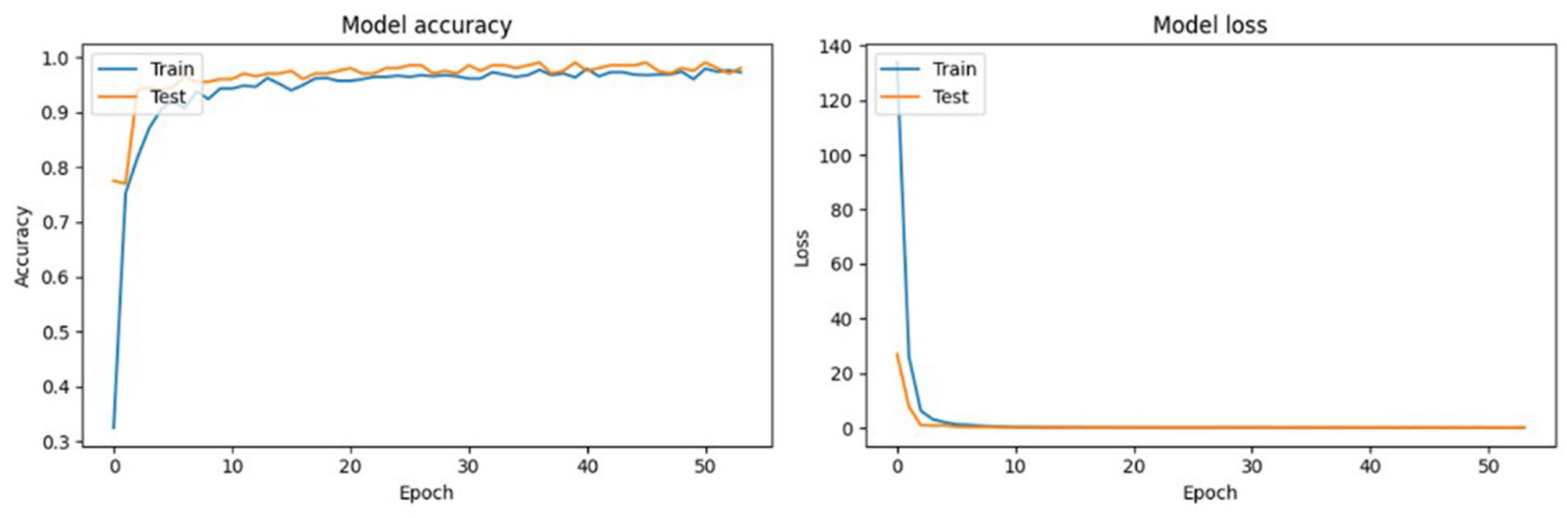
Training and testing performance of the ResNet-101 model over 50 epochs, presented in two plots: Model Accuracy and Model Loss.

VGG-19 was also a top-performing model in this study, characterized by its deep convolutional layers that are able to capture intricate details (Simonyan and Zisserman, 2014). While it requires more computational resources, the depth of its architecture allows for the thorough extraction of features, which is instrumental in distinguishing between classes that share close similarities. VGG-19′s design focuses on increasing the depth with smaller convolution filters, which effectively increases the model's capacity to learn finer details in the Conifer pollen species without substantially widening the network (Simonyan and Zisserman, 2014; Shen et al., 2019). The training and testing performance of ResNet101 over 50 epochs, presented in Figure 5, demonstrates the model's convergence behavior. The accuracy plot indicates that the model achieves high accuracy early in the training process and maintains it throughout. This suggests that ResNet101 efficiently learns to generalize from the training data. The loss plot complements this by showing a rapid decrease in loss during the initial epochs, which stabilizes as training progresses, indicating that the model is effectively minimizing the error.

**Figure 6:**
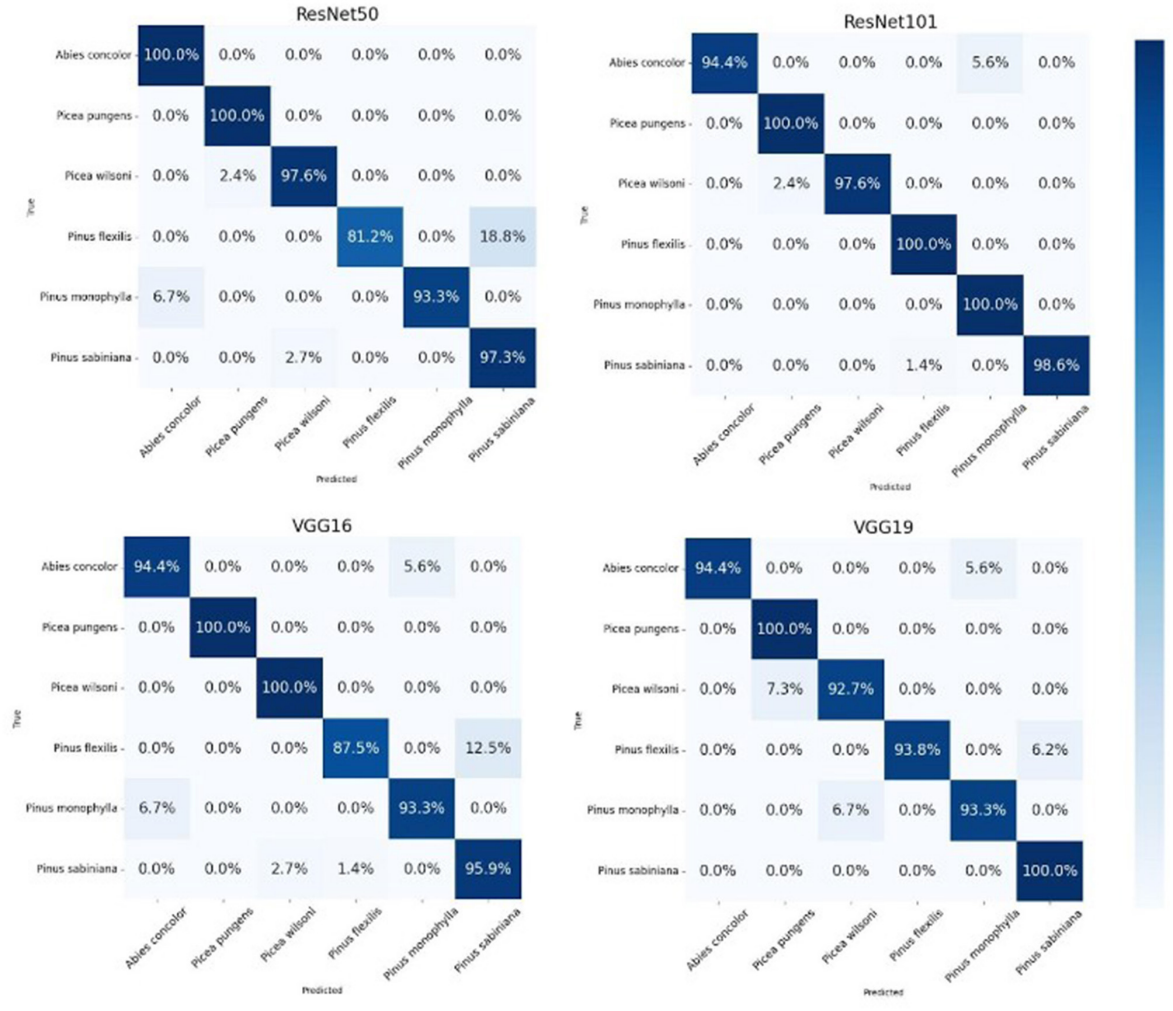
Confusion matrix for the ResNet-101 model illustrating classification results for particulate matter from six tree species.

The confusion matrices for ResNet50, ResNet101, VGG-16, and VGG-19 further illustrate the models' performance by showing the classification results for particulate matter from six tree species (Figure 6). Each cell in the matrices represents the number of predictions made by the models. The diagonal cells indicate correct predictions, while off-diagonal cells represent misclassifications. The high values along the diagonal and low values off the diagonal underscore the models' accuracy in classifying the pollen grains. For instance, the matrices show perfect classification for several species, with only minor misclassifications, demonstrating the models' precision and recall. Our study achieved high accuracy in identifying closely related coniferous species, demonstrating the value of AI in palynology to advance our ability to rapidly identify and distinguish between similar pollen grains. Despite challenges such as extensive training needs and the time-intensive nature of creating training datasets, the integration of AI helps overcome the limitations of traditional methods, which often require meticulous manual effort and are prone to errors, particularly with similar pollen grains. Our models reduce these errors by effectively learning complex patterns and subtle distinctions, speeding up the research process and enabling large-scale analysis that would be impractical manually. This emphasizes the critical role of trained palynologists in ensuring precise image capturing to grow these models on more species in the future.

## 4 Conclusion

This study highlights the benefits of integrating advanced AI technologies, specifically deep learning models like ResNet and VGG, into traditional pollen analysis using light microscopes. While AI applications are powerful, the expertise of trained palynologists remains essential for creating necessary datasets, emphasizing their indispensable role. Applying these models, we achieved high accuracy and efficiency in classifying pollen grains from closely related and hard-to-distinguish coniferous species. Using AI in pollen identification is not without challenges. It requires extensive training and expertise. Capturing high-quality images is important, as they need to be consistently scaled and properly focused, which is time-consuming and demands precision. Despite these difficulties, this research demonstrates the significant advantages of AI in environmental sciences. By merging traditional palynology and ecology research with AI technology, we can use these tools to better understand historical climate patterns, vegetation distributions, and the impacts of environmental changes on human and ecological health. For example, time series exist both in pollen from soil cores and in museum pollen from plant and insect specimens. These time series can be used to reconstruct plant communities (Balmaki et al., 2019; Balmaki and Wigand, 2019), and plant-pollinator interactions (Balmaki et al., 2022a,b, 2024) and then combined with historical climate or disturbance data to test hypotheses of the effects of climate and disturbance on plant communities and interactions. This balanced approach allows us to recognize both the potential and the challenges of using AI, paving the way for more effective and accurate environmental studies.

## Data Availability

The original contributions presented in the study are included in the article/supplementary material, further inquiries can be directed to the corresponding author.
